# Sex-based dimorphism of anticancer immune response and molecular mechanisms of immune evasion

**DOI:** 10.1158/1078-0432.CCR-21-0136

**Published:** 2021-05-20

**Authors:** Fabio Conforti, Laura Pala, Eleonora Pagan, Vincenzo Bagnardi, Tommaso De Pas, Paola Queirolo, Elisabetta Pennacchioli, Chiara Catania, Emilia Cocorocchio, Pier Francesco Ferrucci, Maristella Saponara, Gianmarco Orsolini, Paola Zagami, Eleonora Nicoló, Filippo De Marinis, Giampaolo Tortora, Emilio Bria, Saverio Minucci, Hadine Joffe, Paolo Veronesi, Jennifer Wargo, Rachel Rosenthal, Charles Swanton, Alberto Mantovani, Richard D. Gelber, Giuseppe Viale, Aron Goldhirsch, Giuseppe Giaccone

**Affiliations:** 1 Division of Medical Oncology for Melanoma & Sarcoma, IEO, European Institute of Oncology IRCCS, Milan, Italy; 2 Department of Statistics and Quantitative Methods, University of Milan-Bicocca, Milan, Italy; 3 Division of Thoracic Oncology, European Institute of Oncology, IRCCS, Milan, Italy; 4 Division of Medical Oncology, European Institute of Oncology, IRCCS, Milan, Italy; 5 Department of Experimental Oncology, IEO, European Institute of Oncology IRCCS, Milan, Italy; 6 Comprehensive Cancer Center, Fondazione Policlinico Universitario A. Gemelli, IRCCS, Rome, Italy; 7 Medical Oncology, Department of Translational Medicine and Surgery, Università Cattolica Del Sacro Cuore, Roma, Italy; 8 Harvard Medical School, Boston, Massachusetts. Mary Horrigan Connors Center for Women's Health and Gender Biology, Brigham and Women's Hospital, Boston, Massachusetts; 9 Division of Breast Cancer Surgery, IEO, European Institute of Oncology, IRCCS, Milan, Italy; Faculty of Medicine, University of Milan, Milan, Italy; 10 Department of Surgical Oncology and department of Genomic Medicine MD Anderson Cancer Center, Houston, TX, USA; 11 Cancer Evolution and Genome Instability Laboratory, The Francis Crick Institute, Cancer Research UK Lung Cancer Centre of Excellence, University College London Cancer Institute, University College London, London, UK; 12 Humanitas Clinical and Research Center IRCCS and Humanitas University, Milan; 13 Department of Data Science, Dana-Farber Cancer Institute, Harvard Medical School, Harvard T.H. Chan School of Public Health, and Frontier Science & Technology Research Foundation, Boston. USA; 14 Department of Pathology, IEO, European Institute of Oncology IRCCS Milan, Italy; 15 University of Milan, Milan, Italy; 16 MultiMedica San Giuseppe Hospital, Milan, Italy; 17 Department of Oncology, Weill Cornel Medicine, New York, USA

## Abstract

**Purpose:**

We previously demonstrated that sex influences response to immune-checkpoint inhibitors.

Here we investigate sex-based differences in the molecular mechanisms of anticancer immune-response and immune evasion in patients with NSCLC.

**Experimental Design:**

We analyzed a) transcriptome-data of 2575 early-stage NSCLCs from 7 different datasets; b) 327 tumor-samples extensively characterized at the molecular level from the TRACERx lung study; c) two independent cohorts of respectively 329 and 391 patients with advanced NSCLC treated with anti-PD1/anti-PDL1 drugs.

**Results:**

As compared with men, the tumor microenvironment (TME) of women was significantly enriched for a number of innate and adaptive immune cell-types, including specific T-cell subpopulations.

NSCLCs of men and women exploited different mechanisms of immune evasion.

The TME of females was characterized by significantly greater T-cell dysfunction status, higher expression of inhibitory immune-checkpoint molecules and higher abundance of immune-suppressive cells, including Cancer Associated Fibroblasts, MDSCs and Regulatory T-cells.

By contrast, the TME of males was significantly enriched for a T-cells excluded phenotype.

We reported data supporting impaired neoantigens presentation to immune system in tumors of men, as molecular mechanism explaining the findings observed.

Finally, in line with our results, we showed significant sex-based differences in the association between TMB and outcome of patients with advanced NSCLC treated with anti-PD1/PDL1 drugs.

**Conclusions:**

We demonstrated meaningful sex-based differences of anticancer immune response and immune evasion mechanisms, that may be exploited to improve immunotherapy efficacy for both women and men.

## Introduction

Meaningful differences of both innate and adaptive immune responses between men and women explain different prevalence and mortality from autoimmune and infectious diseases and from several types of cancers.^1,2^ Such sex-based differences of immune responses reflect complex interactions among genes, hormones and environment.^1,2,3^

We demonstrated that patients’ sex influences the response to anticancer immunotherapy.^4,5^

First, we performed a meta-analysis including 20 RCTs comparing immunotherapy-containing regimens to standard treatments in several solid tumors, and we showed that men obtain significant larger survival benefit than women when treated with anti-CTLA4 or anti-PD-1 antibodies as monotherapy.^4^

Subsequently, we showed that women with advanced non-small cell lung cancer (NSCLC) experienced impressive larger survival benefit than men, from the combination of chemotherapy with an anti-PD-1 or anti-PD-L1.^5^

We hypothesized that such heterogeneity of response to different immunotherapy strategies, is due to differences in the molecular mechanisms that drive anticancer immune response in men and women.

To explore such hypothesis, we investigated sex-based differences in key elements of anticancer immune response, in men and women with early non-small cell lung cancer (NSCLC).

## Materials and methods

### Source data

We analyzed datasets containing data on genome-wide transcriptome analysis of NSCLC samples, from the Lung Cancer Explorer (LCE) project.^6^

For our analyses, we focused on the largest datasets on adenocarcinoma and squamous cell carcinoma available in the LCE-project: we included in the analysis all the datasets with data on more than 250 tumor samples and at least 25 samples from female patients for adenocarcinoma and/or more than 100 tumor samples and at least 10 samples from female patients for squamous-NSCLC.

Details on extensive procedures adopted for reprocessing and normalizing expression data, quality control assessment and standardization of the datasets to maximize comparability, has been previously reported. ^6^ More details are also reported in the supplementary methods section.

We also analyzed Whole Exome Sequencing (WES) data of 327 tumor regions from 100 patients with NSCLC and RNAseq data of a subset of 164 tumor regions from 64 patients, included in the TRACERx lung study (https://clinicaltrials.gov/ct2/show/NCT01888601).

More details on the patient cohort enrolled in the TRACERx lung study have been previously reported.^7–9^

Finally, we analyzed data of two independent cohorts of patients with advanced NSCLC treated with anti-PD1 or anti-PD-L1 monotherapy, and for which individual patient data (IPD) were available on tumor mutational burden (TMB) and patients’ outcome. ^10–11^

These two cohorts were represented respectively by 1) patients included in the MSKCC database and 2) patients enrolled in the POPLAR and OAK randomized clinical trials (RCTs).^10–11^

More details on these two patients’ cohorts have been previously reported.^10–11^

### Computational and statistical analyses

#### 1 Assessment of sex-based differences in the tumor microenvironment (TME) of cell type composition of the immune infiltrate and expression levels of immune-related pathways and immune checkpoint molecules

Gene-expression data were analyzed through the previously validated xCell algorithm, to estimate the abundance of 64 different cell types in the microenvironment of each tumor sample included in the datasets of the LCE-project as well as of the TRACERx lung study.

The entire pipeline of xCell has been previously described ^12,13^, and more details are reported in the supplementary methods sections.

For each single dataset, mean values of enrichment score (ES) for the 64 different cell-types were calculated in tumors of men and women and then compared using a multivariable linear regression model adjusted for patient age, stage at diagnosis, tumor-histotype, smoking status.

We then performed a meta-analysis of the adjusted sex-related differences obtained in each single dataset using a random-effects model. The false discovery rate (FDR) was used to correct for multiple comparisons. Pooled estimate higher than 0 indicated a greater ES in females, and lower than 0 a greater ES in males.

Gene-expression data from the LCE-project datasets were analyzed through the Gene Set Enrichment Analysis method proposed by Subramanian et al.^13^, using the following gene sets (GSs) collections:

1) C5 collection of the Molecular Signatures Database (MSigDB) v6, that includes GSs derived from Gene Ontology terms, which allows to comprehensively assess all biological processes, molecular functions and components of cells.
2) 16 specific gene signatures, recently defined through single-cell RNA sequencing characterization of the T-cells landscape of NSCLC. Each GS is associated with a different T-cell subpopulation, characterized by specific functional state and phenotype, including CD8+ and CD4+ naïve T-cells, effector T-cells, pre-exhausted and terminally exhausted T-cells, and T-cell subpopulations with intermediate functional states as well as T regulatory Cells (supplementary table 1).^14^
3) Two different, previously validated signatures including 26 and 24 genes upregulated in hypoxic TME (supplementary table 1). ^15–17^

A pooled estimate of the Normalized Enriched Score (NES) for each Gene Set analyzed, was obtained meta-analyzing the LCE-project datasets, as described in details in the supplementary methods sections.

A pooled NES higher than 0 indicated a greater enrichment of the gene set in females, and lower than 0 a greater enrichment in males. The false discovery rate (FDR) was used to correct for multiple comparisons

Finally, a curated list of 78 genes with a key role in anticancer immune response was derived from Thorson et al^18^, and the expression levels of each gene were evaluated to assess differences between tumors of male and female patients in each single dataset using a multivariable linear regression model adjusted for patient age, stage at diagnosis, tumor-histotype and smoking status.

We then performed a meta-analysis of the adjusted sex-related differences obtained in each single dataset using a random-effects model and we corrected for multiple comparisons with FDR.

Only one of these 78 genes is located on sex chromosomes (i.e., CD40LG gene located on X chromosome)

#### 2 Assessment of sex-based differences in mechanisms of immune evasion

Gene-expression data from LCE-project datasets were analyzed through the validated Tumor Immune Dysfunction and Exclusion (TIDE) tool, that permits quantification of the activation status of two major mechanisms of immune-evasion exploited by tumors: the induction of T-cell dysfunction (T-cell dysfunction mechanism) and the inhibition of T-cell infiltration into TME (T-cell exclusion mechanism).^19^

For each tumor sample we calculated two scores, the “T-cell dysfunction score” and “T-cell exclusion score”: both scores range from -4 to +4, with the higher score levels being associated with greater activation status of the corresponding mechanism of immune-evasion.^19^

For each single dataset, mean values of the “T-cell dysfunction score” and “T-cell exclusion score” were calculated in tumors of men and women and then compared using a multivariable linear regression model adjusted for patient age, stage at diagnosis, tumor-histotype and smoking status.

We then performed a meta-analysis of the adjusted sex-related differences obtained in each single dataset using a random-effects model. The Q test was performed to assess between-study heterogeneity, and the I^2^ statistics, which express the percentage of the total observed variability due to heterogeneity, were also calculated

A pooled-estimate score higher than 0 indicated a greater activation status in females of the corresponding mechanism of immune-evasion, and lower than 0 a greater activation in males.

#### 3 Assessment of sex-based differences in TCR repertoire diversity, tumor neoantigens load and alterations in neoantigens presentation machinery

Multiregion Bulk RNAseq and WES data from TRACERx lung study were employed to assess the following key elements of the immune response in each tumor, as previously described:

a) T-cell receptor abundance and entropy score. ^20,21^
b) amount of ubiquitous expanded TCRs ^8,9^
c) number of predicted tumor neoantigens and their clonal and subclonal distribution ^7,22,23^
d) occurrence of loss of heterozygosity (LOH) events at the HLA class I locus ^8^
e) occurrence of genetic disruptive events (i.e. non-silent mutations or copy-number loss defined relative to ploidy) in antigen presentation pathway genes, including: CIITA, IRF1, PSME1, PSME2, PSME3, ERAP1, ERAP2, HSPA (also known as PSMA7), HSPC (also known as HSPBP1), TAP1, TAP2, TAPBP, CALR, CNX (alias CANX), PDIA3 and B2M. ^8,24^

Differences between tumors of male and female patients were assessed using a multivariable linear regression model adjusted for patient age, stage at diagnosis, tumor-histotype and smoking status, and we corrected for multiple comparisons with FDR.

More details on analyses performed are reported in the supplementary methods section.

#### 4 Evaluation of sex-based differences in the association between Tumor Mutational Burden (TMB) and outcome of patients treated with anti-PD1 or anti-PDL1 drugs

We analyzed data from the MSKCC dataset on patients treated with anti-PD1 or anti-PD-L1 drugs, to assess the association between tissue-based TMB (tTMB) and OS according with patients’ sex.

Data of patients treated with the anti-PDL1 atezolizumab in the OAK and POPLAR RCTs, were evaluated to assess the association between blood-based TMB (bTMB) and PFS according with patients’ sex.

We did not assess sex-based differences in the association between bTMB and OS, since in the original analysis performed on the whole OAK and POPLAR patients population, a significant predictive value of bTMB was reported for PFS but not for OS.^11^

For all the analyses reported, the tissue and blood TMB were analyzed as continuous variables. Wilcoxon rank-sum test was used to compare the distribution of tissue or blood TMB between female and male patients. Cox proportional hazard regression model was used to evaluate the association between tTMB and patient OS as well as between bTMB and patient PFS. Male and female subgroups were analyzed separately.

Departure from linearity in the relationship between tissue or blood TMB and the hazard of death or progression, was investigated with restricted cubic spline (RCS) models with four knots located at the 20^th^, 40^th^, 60^th^ and 80^th^ percentiles of the TMB distribution of female and male patients, respectively.^25^ The likelihood ratio test was used to determine whether the RCS model significantly increased the likelihood function compared with a simpler model that assumed a linear relationship.

Multivariable analyses were performed excluding patients with tumors harboring EGFR gene mutation or ALK gene translocation and considering only those patients with available data on the following adjustment factors: age, smoking history, tumor histotype, type of specimen analyzed, number of metastatic sites at enrollment, sum of longest diameter of target lesions at baseline, and PD-L1 expression levels.

Statistical analysis were performed with SAS software v. 9.4 (SAS Institute, Cary, NC) and R software (version 3.4.1).

Analyses conducted on the TRACERx dataset included the first 100 patients prospectively analyzed by the lung TRACERx study (https://clinicaltrials.gov/ct2/show/NCT01888601), that was approved by an independent Research Ethics Committee (13/LO/1546), and conducted in accordance with Declaration of Helsinki, obtaining written consent from all the subjects enrolled. All the other analyses were meta-analyses of published and public available data.

## Results

### 1 Sex-based differences in the tumor immune infiltrate

Seven datasets of the LCE-project fulfilled the inclusion criteria and were included in the analysis.^26–32^

Totally, 2575 tumor samples were analyzed: 1528 tumors (59.3%) were from men and 1047 (40.7%) from women (table 1).

732 tumors (28.4%) were from patients younger than 60 years, 934 (36.3%) from patients aged between 61 and 70 and 853 (33.1%) from patients older than 70 years; 1880 patients (73.0%) were current or former smokers, 206 (8.0%) non-smokers, and for 489 patients (19.0%) the smoking history was unknown.

The majority of patients (i.e., 95.9%) had stage I-III tumors, and only 62 (2.4%) had a disease in stage IV.

Five datasets reported data of 1967 (76%) adenocarcinoma (i.e. TCGA-LUAD, Schabath et al, Roussueax et al, Schedden et al and Sato et al.)^30–32^, and 2 datasets of 608 (24%) squamous-NSCLCs (i.e. TCGA-LUSC and Noro et al.).^26,27^

Out of 1967 adenocarcinoma tumors, the EGFR and ALK mutational status was respectively known for 717 (36.4%) and 245 (12.4%) cases.

Tumor samples harboring EGFR or ALK alterations were respectively 127 (17.7 %) and 34 (13.9 %).

Tumor samples from 100 patients from the TRACERx lung study were also studied. ^7–9^

The cohort consisted of 62 men and 38 women, with a median age of 68. Eighty-eight patients were current or former smokers and only 12 patients were non-smokers. Sixty-one tumors were adenocarcinoma, 32 squamous-cell carcinoma, and 7 were classified as other histology.

The cohort was predominantly early-stage: I (62), II (24), IIIa (13), IIIb (1). Totally, 327 tumor regions (323 primary tumor regions and 4 lymphnode metastases) were analyzed.

We assessed differences in the cell-type composition of the immune infiltrate between tumors of male and female patients.

**Figure 1:**
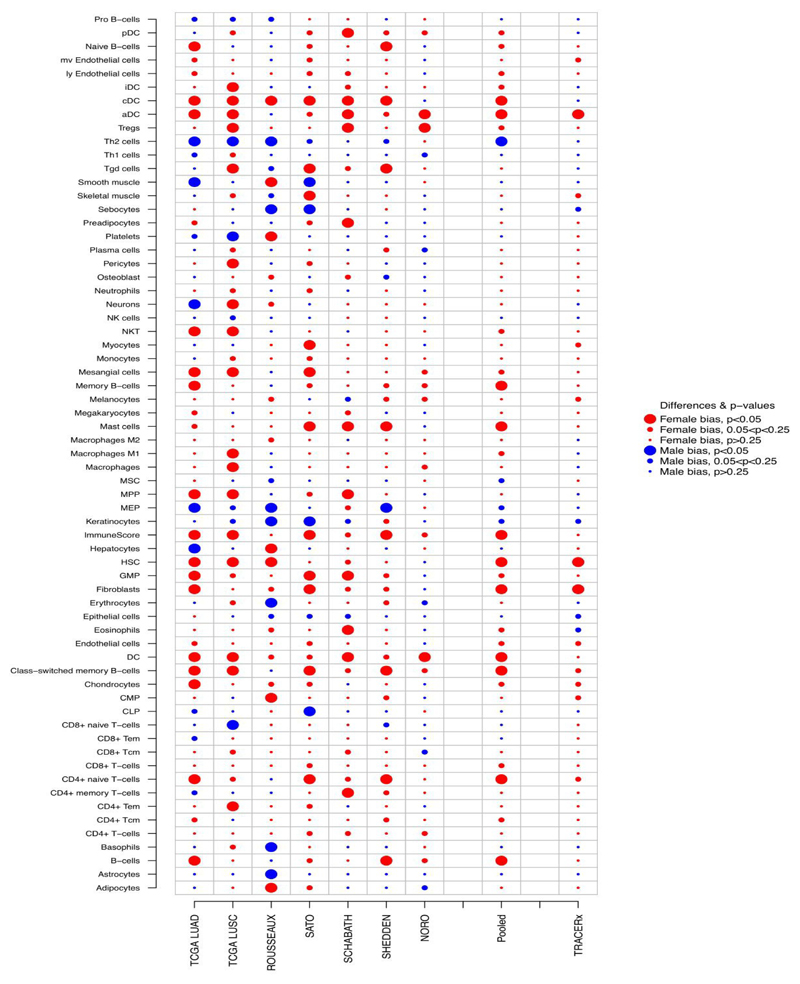
Summary plot of sex-based differences in the cell type composition of the immune-infiltrate. Figure shows, for each of the 7 LCE-project dataset, p- values for the difference between the Enrichment Score (ES) of female tumors and male tumors based on a multiple linear regression model adjusted by age, smoking status, tumor-histotype and. Pooled p-values were calculated using random-effects models and corrected for FDR. Female bias (legend) stands for immune-cell types found enriched in the tumor microenvironment of female patients as compared with males (i.e., estimates for the difference in ES higher than 0); male bias (legend) stands for immune-cell types found enriched in the tumor microenvironment of male patients (i.e., estimates for the difference in ES lower than 0). For the TRACERx dataset p-values for the difference between the ES of female tumors and male tumors based on a linear regression model were reported.

Figure 1 shows sex-based differences in the abundance of immune cells found in each of the 7 LCE-project datasets as well as the pooled meta-analytic results.

In the pooled analysis, the innate and adaptive immune cells found enriched in the TME of women as compared with men, at a FDR cut-off ≤0.05 were (fig. 1):

1) Dendritic cells (including plasmocytoid dendritic cells, conventional dendritic cell, and activated dendritic cells);
2) CD4+ T-cells (including CD4+ naive T-cells and CD4+ central memory T-cells);
3) B-cells (including Memory B-cells and Class-switched memory B-cells);
4) Mast-cells.

Innate and adaptive immune cells found enriched in the TME of female patients, at FDR cut-off ≤0.25 were (fig. 1):

1) Regulatory T-cells;
2) Natural killer T-cells;
3) Macrophages M1-type;
4) CD8+ T-cells;
5) Eosinophils;

The TME of female patients, was also significantly enriched in cancer-associated fibroblasts (CAFs; FDR=0.09), hematopoietic stem cells (HSCs; FD=0.09) and Granulocyte-Macrophage progenitors (GMPs; FDR=0.09), that are respectively mesenchymal and myeloid-derived cells known to exert immunosuppressive activity in TME (fig. 1).^33,34^

The only immune cell type found significantly enriched in the TME of men at FDR =0.09, was type 2 T-helper cell.

Analysis of tumors from the TRACERx lung study cohort, confirmed a significant enrichment of activated dendritic cells, CD4+ naïve T-cells, HSC and CAFs in TME of women (fig. 1). Furthermore, a trend for enrichment in the TME of women was also observed for CD4+ central memory T-cells (p=0.1) and CD8+ T-cells (p=0.1; fig.1).

In line with these data, showing a more abundant immune infiltrate in tumors of women, GSEA of the LCE-project datasets using the C5 MSigDB collection revealed that among the top 1% gene sets (GSs) significantly enriched in the TME of women as compared with men (FDR<0.05) and ranked accordingly to the NES, the large majority were GSs directly related to immune responses. (supplementary fig. 1 shows the top 1% GSs enriched in women and men; supplementary table 2 reports GSEA results for all the 5917 GSs analyzed)

Most of the immune-related GSs found significantly enriched in women, concerned the regulation of leukocytes – including T-cells – proliferation, activation and cytotoxicity, regulation of cytokine secretion and signaling, and response to Interferon I and gamma pathways. (supplementary fig. 1)

Another group of GSs significantly enriched in tumors of women, were related to leukocyte cell-cell adhesion and migration, and indeed we found that TME of women was characterized by significantly higher expression levels of a number of chemokines, receptors and integrins specifically known to play a key role in leukocytes extravasation and tumor infiltration, including: CCL5, CX3CL1, CXCL9, BTN3A2, ICAM1 and LFA1 (FDR≤0.05 for each with exception of CXCL9 for which FDR was 0.06 supplementary fig. 2).^18,33,34^

Notably, none of the top 1% gene sets found significantly enriched in tumors of men and ranked accordingly to the NES, were related to immune responses, while the large majority were related to DNA replication and repair mechanisms (supplementary fig. 1)

### 2 Sex-based differences of the T-cells landscape

We assessed the intratumor abundance of specific T-cells subpopulations, previously identified in TME of patients with NSCLC, and characterized by different functional state and phenotype.^14^

**Figure 2:**
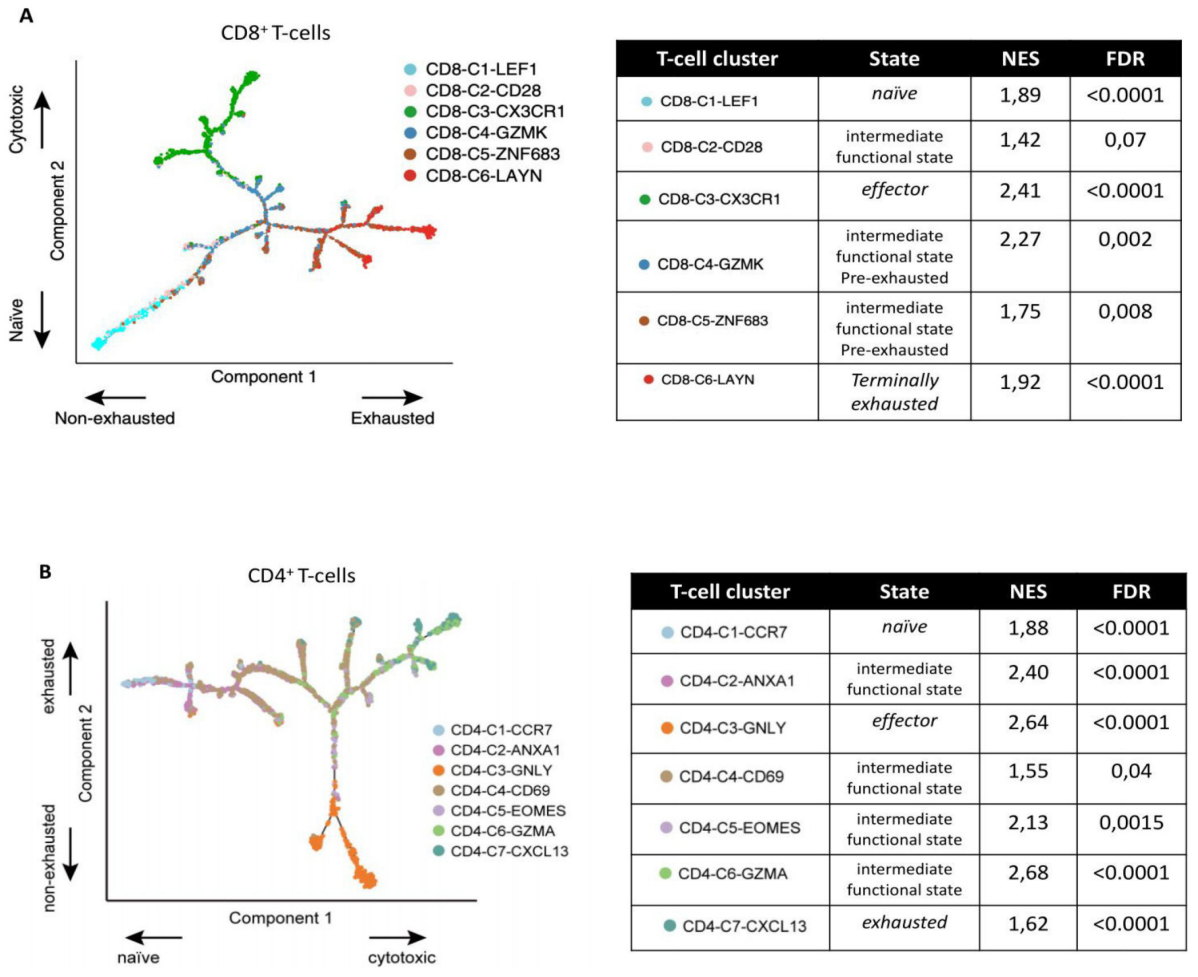
Sex-based differences in abundance of specific CD8+ (panel A) and CD4+ (Panel B) T-cell subpopulations. Figures in panel A and B, show respectively the branched trajectory of CD8+ T and CD4+ T cells state transition in a two-dimensional state-space as described in Guo et al. Each dot corresponds to a T-cell subpopulation, colored according to its cluster label. Arrows show the increasing directions of certain T cell properties. Tables in panel A and B report sex-based difference in abundance of each specific CD8+ T and CD4+ cell subpopulation: NES >0 indicates enrichment in tumors of women, NES<0 in tumors of men

All the T-cell subpopulations analyzed, were significantly enriched in TME of women, including (supplementary table 3):

1) CD8+ and CD4+ naïve T-cells (i.e. respectively, CD8-C1-LEF: NES=1.89, FDR<0.0001, fig. 2A; and CD4-C1-CCR7: NES=1.88, FDR <0.0001, supplementary fig. 2B);
2) CD8+ and CD4+ effector T-cells, (i.e. respectively, CD8-C3-CX3CR1: NES=2.41, FDR<0.0001, fig. 2A; and CD4-C3-GNLY: NES:2.64, FDR<0.0001, fig. 2B)
3) CD8+ and CD4 T-cell subpopulations with an intermediate functional state (i.e. CD8-C2-CD28: NES=1.42, FDR=0.07, fig. 2A; CD4-C2-ANXA1: NES=2.4, FDR<0.0001, fig. 2B)

Taken together these data demonstrate meaningful sex-based difference of the T-cell driven antitumor immune response, that was further supported by results of TCR analysis.

Previous works demonstrated that a higher clonality of TCR-repertoire of tumor infiltrating lymphocytes (TILs), is a proxy for T-cell immune response against tumor antigens, as compared with a polyclonal TCR-repertoire.^8,9,35^

**Figure 3:**
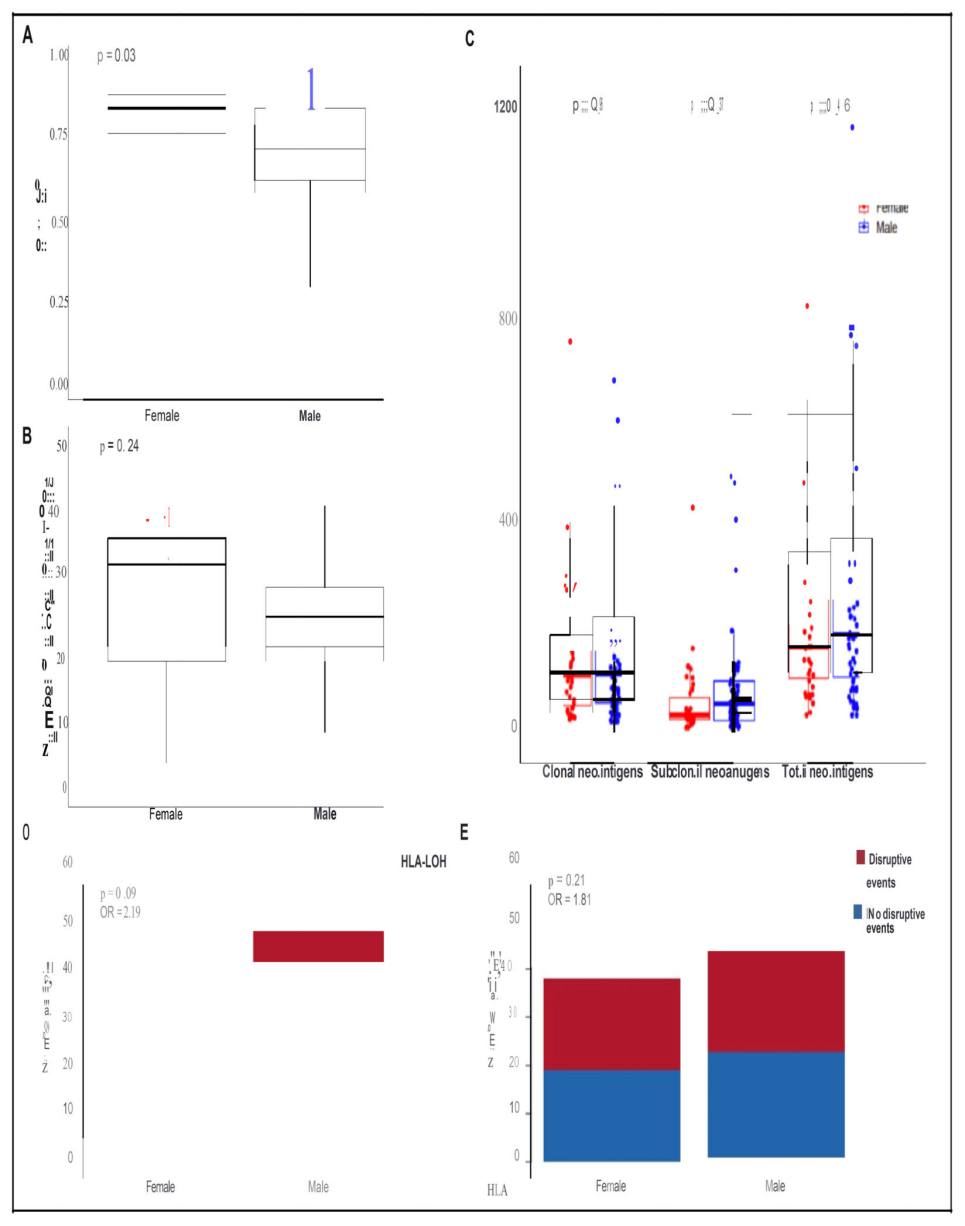
Sex-based differences in TCR repertoire, tumor antigenicity and alterations in antigen presentation machinery. Figure reports TCR diversity score (**panel A**), number of expanded ubiquitous TCRs (**panel B**), clonal, subclonal and total number of neoantigens (**panel C**) calculated in tumors of men and women. **Panel D** and **panel E** report respectively, number of patients with tumors harboring or not HLA type I Loss of Heterozygosity (LOH), or disruptive events in other genes of antigens presentation machinery

Analysis of the TCR-repertoire of multiregion tumor samples from TRACERx lung study cohort, showed a significantly greater TCR clonality in TILs of women (median TCR entropy score 0.83 in females versus 0.67 in males, p=0.03; fig. 3A).

Coherently, we also found a numerically higher amount of “expanded ubiquitous TCRs”-i.e. TCRs expanded AND present in all tumors regions assessed in multiregional tumor analysis-in TME of women as compared with men, albeit this was not statistically significant (the median number of ubiquitous expanded TCRs was 33 in females and 24 in males, p=0.24; number of patients analyzed: 14 females and 25 males. fig 3B).

### 2 Sex-based differences in mechanisms of immune-evasion

To assess sex-based differences in mechanisms exploited by tumors to evade immune response, we analyzed the 7 datasets of LCE-project through the TIDE tool.^19^

**Figure 4:**
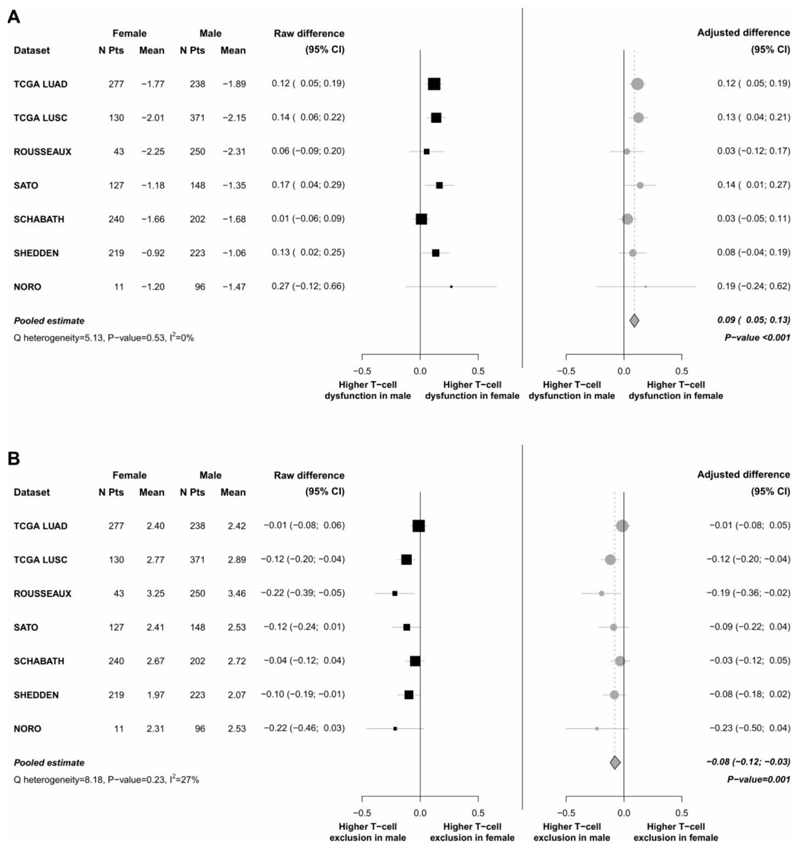
Meta-analysis of differences in the “T-cell dysfunction score” (Panel A) and “T-cell exclusion score” (Panel B) assessed in tumors of female and male patients. For each dataset the mean values of “T-cell dysfunction score” (panel A) and “T-cell exclusion score” (panel B) are calculated separately in tumors of male and female patients, and the respective raw and adjusted (by age, smoking status, tumor-histotype and stage) differences are presented, as well as the meta-analytic pooled estimates calculated using a random effects model.

In all the 7 datasets, the mean value of the “T-cell dysfunction” score was higher in tumors of women as compared with men. The pooled adjusted difference estimate was 0.09 (95%CI, 0.05 to 0.13, p<0.001), confirming significantly greater T-cell dysfunction status in tumors of women (fig. 4A).

On the contrary, the mean value of the “T-cell exclusion” score was always higher in tumors of men. The pooled adjusted difference estimate was -0.08 (95%CI, -0.12 to -0.03; p=0.001), confirming significantly greater activation status of such mechanism of immune-evasion in tumors of men (fig. 4B).

Consistently with the higher T-cell dysfunction score found in TME of women, we demonstrated a significantly higher abundance in women of CD8+ and CD4+ T-cells subpopulations with both a pre-exahusted phenotype (i.e., CD8-C4-GZMK: NES=2.27, FDR=0.002;

CD8-C5-ZNF683: NES=1.75, FDR=0.008; fig. 2A) and a terminally exhausted phenotype (i.e., CD8-C6-LAYN: NES=1.92, FDR<0.0001, fig. 2A; and CD4-C7-CXCL13: NES=1.62, FDR<0.0001, fig. 2B)

We also found a significantly higher abundance of a specific subpopulation of T regulatory cells in TME of women (i.e., CD4-C9-CTLA4 [TNFRSF9-]: NES=2.31, FDR<0.0001; supplementary table 3)

Notably, there was a significantly higher expression levels of a number of inhibitory immune-checkpoints in TME of females (including TIM3, TIGIT, BTLA, IDO1, ADORA2A, ENTPD1, BTN3A1, TNFRSF14, and VISTA at FDR≤0.05 and LAG3 at FDR=0.09), which are known to play a key role in T-cells exhaustion mechanisms and are currently explored as therapeutic targets (Supplementary fig. 2).^18,33,34^

The lower abundance of a number of immune cell types in TME, the significantly higher T-cell exclusion score, the smaller TCR repertoire clonality and the lower amount of ubiquitous expanded TCRs observed in tumors of men, are all elements indicating a less efficient tumor recognition and infiltration by immune-system.

The main molecular mechanisms that have been showed to impair infiltration of TME by immune system, and underlie the immune-excluded phenotype, include aberrant activation of the TGF-β or WNT/β-catenin pathways, dysfunctional metabolic conditions of the TME such as a high degree of hypoxia, low tumor neoantigens load and/or alterations in tumor antigen presentation mechanisms.^33,34,36^

We thus explored the hypothesis that one or more of these mechanisms could explained the lower degree of immune infiltration and the enrichment of the T-cells excluded phenotype observed in the TME of men as compared with women.

We found no sex-based differences in the activation status of the TGF-β or WNT/β-catenin pathway, in all the 7 LCE-project datasets (data not shown).

As compared with women, TME of men was characterized by a higher degree of hypoxia, as revealed by significant enrichment of two different hypoxia gene signatures (Boffa et al Gene Set^15^: NES= -2.37, FDR<0.001; Yang et al GS ^16^,^17^: NES= -2.33, FDR<0.001; supplementary table 3)

Notably, the expression levels of VEGFA was significantly higher in TME of men (FDR<0.25, Supplementary fig. 2)

Tumors from the TRACERx cohort were analyzed, to estimate sex-based differences in the predicted tumor neoantigens load and/or in the neoantigens clonal distribution.

There was no sex-based difference in the total number of predicted tumor neoantigens, nor in the number of clonal (i.e. shared by all cancer cells) or subclonal (i.e. carried by a fraction of the cancer cells population) neoantigens.(fig. 3C)

Finally we used data from both the TRACERx lung study cohort and the LCE-project datasets, to explore the hypothesis that tumors from men had impaired antigen presentation mechanisms as compared with women.

As hypothesized, we found significantly lower expression levels of both HLA Class I and Class II molecules in the TME of men as compared with women (FDR ≤0.05; Supplementary fig. 2). GSEA showed that “MHC protein complex”, “peptide antigen binding” and “b2microglobulin binding” were among the top 1% gene sets significantly enriched in TME of women as compared with men, (FDR ≤0.05; supplementary fig. 1). There also was a borderline significantly higher frequency of loss of heterozygosity (LOH) events at the HLA class I locus in tumors of men (Odds Ratio for HLA-LOH events in tumors of men versus women: 2.19; p=0.08; fig. 3D) as well as a numerically higher frequency for all other genetic disruptive events in genes involved in tumor antigen presentation mechanisms, albeit this was not statistically significant (Odds Ratio for genetic disruptive events in tumors of men versus women: 1.81 p=0.2; fig. 3E)

### 3 Sex-based differences in the association between TMB and patients outcome

Since we found that tumors of men had impaired neoantigens presentation mechanisms as compared with women, we analyzed data of the cohort of 329 patients from MSKCC dataset, with advanced NSCLC and treated with anti-PD-1 or anti-PD-L1 as monotherapy, to test the hypothesis of sex-based differences in the association between tTMB and patients outcome.^10–11^

We analyzed data from 167 women and 162 men. During a median follow-up of 9 months (IQR 3-18 months), 99 and 111 deaths occurred in women and men, respectively. The median tTMB value was 7.9 mutations/Megabase (Mb) in female (min-max range 0-55, IQR 3.9-12.3) and 6.9 mutations/Mb in males (min-max range 0-100, IQR 4.4-12.8; Wilcoxon p-value = 0.98).

A higher tTMB was associated with improved overall survival in both, women (OS-HR for increase of 10 unit of tTMB/Mb: 0.72, 95% CI 0.54-0.95; p-value=0.02) and men (OS-HR for increase of 10 unit of tTMB/Mb: 0.76, 95% CI 0.62-0.94; p-value=0.01).

However, there was a significantly sex-based difference in the linearity of the association between tTMB and patients OS.

**Figure 5:**
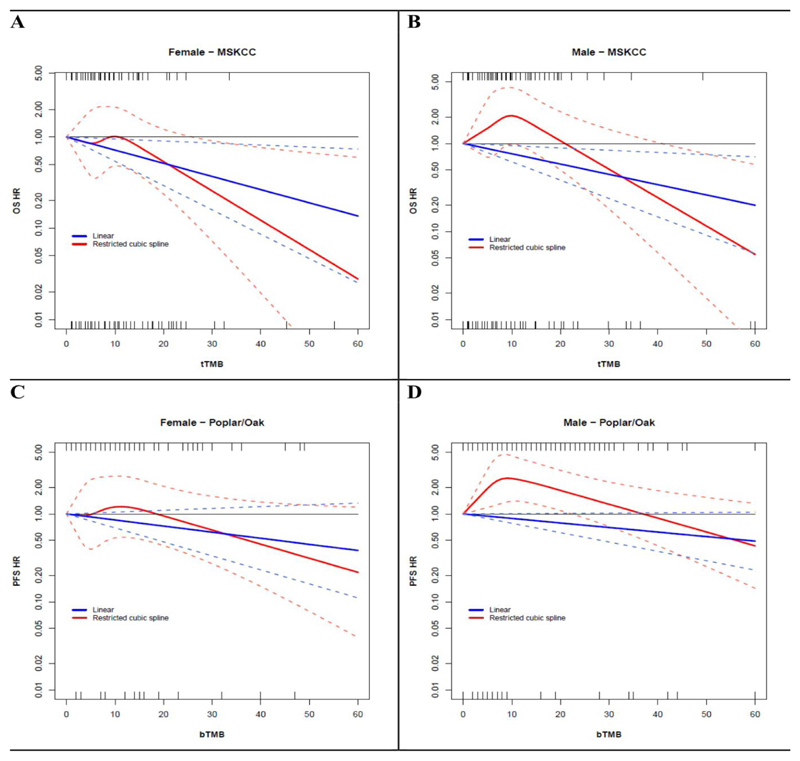
OS and PFS by TMB and gender. **Panel A and B** show the dose-response association between tissue-TMB (treated as a continuous variable) and the hazard of death (OS-HR), assessed respectively in women and in men. Continuous blue and red lines indicate associations modelled through linear versus a restricted cubic spline (RCS) models, respectively. Dotted lines represent 95% confidence intervals. Rug plots for the distribution of TMB in patients with the event (death) and without the event (alive) are reported in the top and in the bottom of the graph, respectively. **Panel C and D** show the adjusted dose-response association between blood-TMB (treated as a continuous variable) and the hazard of disease progression or death (PFS-HR), assessed respectively in women and in men. Adjustment factors were age, tumor histology, number of metastatic sites at enrollment, sum of longest diameter of target lesions at baseline, smoking history and PD-L1 expression levels. Continuous blue and red lines indicate associations modelled through linear versus a restricted cubic spline (RCS) models, respectively. Dotted lines represent 95% confidence intervals. Rug plots for the distribution of TMB in patients with the event (progressed) and without the event (not progressed) are reported in the top and in the bottom of the graph, respectively

A linear trend toward decreasing HR of death for progressively increasing tTMB values was observed in women, along the entire range of TMB values (test for linearity p-value=0.26; fig. 5A), whereas the association between tTMB and OS was not linear in men (test for linearity p-value=0.006, fig 5B).

Results from spline regression analyses suggested an OS advantage starting from tTMB values >10 mutations/Mb in women (fig. 5A), while in males appeared only for higher tTMB values (i.e., TMB>20 mutations/Mb, fig. 5B).

IPD on age, smoking history, tumor histotype and type of specimen analyzed, were available for 262 patients.

224 out of 262 patients-respectively 112 women and 112 men-had an EGFR and ALK wild type tumor, and were further analyzed through multivariable analyses.

Adjusting for age, smoking history, tumor histotype and type of specimen analyzed, we confirmed that tTMB retained a significant linear association with better OS in women (adjusted OS-HR for increase of 10 unit of tTMB/Mb: 0.58, 95% CI 0.37-0.90; p-value=0.02) but not in men, where the association became much less strong and not significant (adjusted OS-HR for increase of 10 unit of tTMB/Mb: 0.81, 95% CI 0.601.08; p-value=0.15).

To confirm significantly sex-based difference in the linearity of the association between TMB and patients outcome, we further analyzed data from 391 patients (128 women and 263 men), with EGFR and ALK wild-type advanced-NSCLC treated with the anti-PDL1 atezolizumab in the OAK and POPLAR RCTs.^11^

During a median follow-up of 11 months (IQR 5-20 months), 110 and 236 PFS events occurred in women and men, respectively. The median blood TMB value was 6.0 mutations in female (min-max range 0-49, IQR 3.0-14.5) and 9.0 mutations in males (min-max range 0-67, IQR 5.0-17.0; Wilcoxon p-value = 0.002).^11^

Adjusting for age, smoking history, tumor histotype, number of metastatic sites at enrollment, sum of longest diameter of target lesions at baseline and PD-L1 expression levels, we confirmed a significant sexbased difference in the linearity of the association between bTMB and PFS.

A linear trend toward decreasing HR of PFS for progressively increasing bTMB values was observed in women, along the entire range of bTMB values (test for linearity p-value=0.34; fig. 5C), whereas the association between bTMB and PFS was not linear in men (test for linearity p-value <0.001, fig. 5D).

Results from spline regression analyses suggested a PFS advantage starting from TMB values >20 mutations in women (fig. 5C), while in males appeared only for higher TMB values (i.e., TMB>37 mutations, fig 5D).

## Discussion

**Figure 6:**
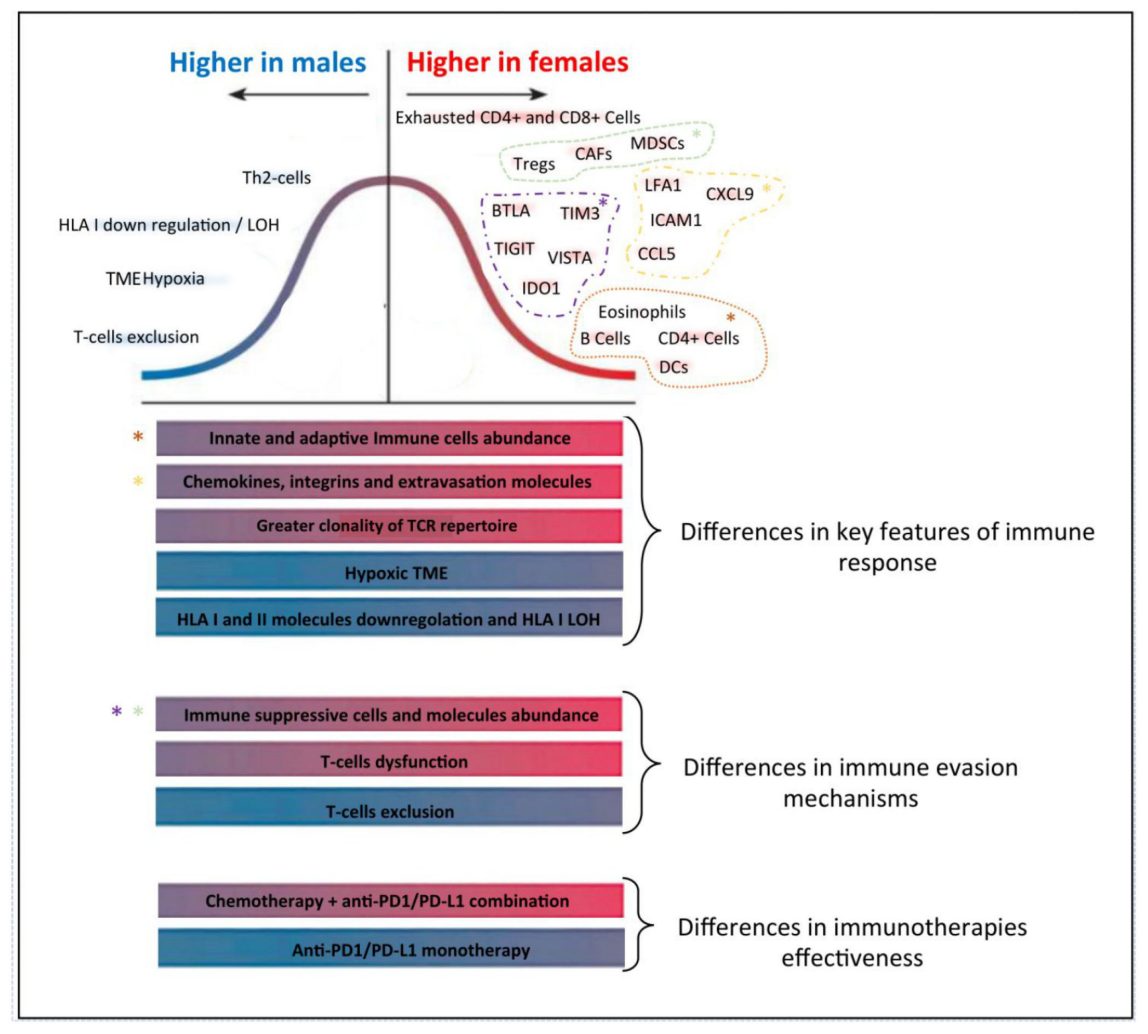
Sex-based differences of molecular mechanisms of anticancer immune response and immune evasion. Figure shows sex-based differences in key features of anticancer immune response and in mechanisms exploited by tumors to evade immune system, described in this work, as well as differences in effectiveness of different immunotherapeutic strategies, demonstrated in previous works. Features explored are represented as bars, and colors indicate their higher prevalence/enrichment in females (red bars) or in male patients (blue bars). The bell curve represents sex-based dimorphism of specific elements of each of the features explored: elements with higher prevalence/enrichment in females are represented in red and on the right of the bell curve, while elements with higher prevalence/enrichment in males are represented in blue and on the left.

Taken together our results, show meaningful sex-based differences in the cell-type composition of the immune infiltrate of patients with NSCLC, including the T-cells landscape, as well as in mechanisms exploited by tumors to evade immune response (summarized in fig.6).

Importantly, we showed that such differences are not related to other variables potentially associated with sex such as age, stage of disease, tumor histotype and smoking status.

On average women mount stronger and more structured immune-response against NSCLC, as highlighted by the higher intratumor abundance of plasmacytoid and activated dendritic cells, CD4+ and CD8+ effector T-cells, memory CD4+ T-cells and B-cells including Class-switched memory cells, as well as by greater clonality of the TCR-repertoire.^33,34,36^

To evade such more efficient initial immune recognition and response, NSCLC arising in women develop more complex and redundant mechanisms of resistance, as revealed by the higher expression of multiple immune-checkpoint molecules with inhibitory functions, as well as by the higher abundance of immune-suppressive cells in the TME, such as CAFs and MDSCs, and Tregs.^33,34,36^

These findings could explain the observed greater dysfunction status of T-cells infiltrating the TME of NSCLC of females, revealed by TIDE and by higher abundance of specific CD4+ and CD8+ subpopulations with a terminally exhausted phenotype.

Notably, it has been shown that the TIDE dysfunction signature specifically reflects the profile of dysfunctional T-cells strongly resistant to ICIs reprogramming.^19^

On the contrary, the TME of NSCLC arising in males was characterized by lower abundance of a number of innate and adaptive immune cell types and by a T-cells excluded phenotype.

We found that such poorer immune infiltration of tumors of men, could depend on a less efficient tumor neoantigens presentation to the immune-system, due to lower expression levels of HLA class I and II molecules and higher frequency of HLA type I LOH events.

Another mechanism underlying the lower immune infiltration of tumors of men, was the higher degree of hypoxia in TME, that has been reported to impair infiltration and proliferation of immune cells.^33,34,36^ Furthermore, the oxidative metabolic state of cancer cells directly affects antigen presentations mechanisms: it has been demonstrated that tumors characterized by higher glycolysis/OXPHOS ratio had significantly lower expression of multiple members of the antigen processing and presentation machinery, including MHC molecules.^37,38^

Importantly, both the hypoxia gene signatures used in our analyses, included biologically relevant genes that map to a set of well-known hypoxia-regulated biochemical pathways, such as glycolysis and gluconeogenesis, lipid metabolism, pH regulation and angiogenesis.^15–17^ Furthermore, we selected two hypoxia signatures that do not overlap (i.e., they share only three genes), have been both previously validated in several independent datasets of different cancer types, and demonstrated to be significantly associated with poorer prognosis in patients with several solid tumors, including NSCLC. ^15–17^

It should be noted that although hypoxia is one of the most important features of the metabolic status of TME affecting immune response, a number of other elements of metabolism are implicated in the modulation of the immune system and may differ according to gender. ^33,34,36^

The selectivity of this our analysis is of course a limitation, and further studies are needed to comprehensively characterize sex-based differences in the metabolic status of the TME and their effects on anticancer immune responses.

We previously demonstrated significant sex-based heterogeneity of response to different type of immunotherapy strategies in patients with advanced NSCLC.^4,5^

The sex-based dimorphism in key elements of anticancer immune response and in mechanisms of tumor immune-evasion showed here, could partially explain our previous observations.

For example, the higher abundance of MDSC, CAF and Tregs found in TME of females, could explain both, the smaller survival benefit experienced by women when treated with anti-PD1 as monotherapy-since it has been recurrently reported that these immune suppressive cells play a major role in ICIs resistance^33,34^-and also the impressively larger survival benefit observed in women treated with the combination of anti-PD1/PD-L1 with chemotherapy-considering the ability of chemotherapy to target these suppressive cell types.^39–40^

It should be noted that our previous works showing sex-based heterogeneity of ICIs efficacy were conducted on patients with advanced NSCLC, while here we analyzed early-stage tumors, and thus other potential molecular mechanisms underlying the sex-based difference of ICIs efficacy that arise later during tumor progression might have been missed by this our work.

Finally, we provided a clear example of the direct clinical implications of our findings, showing meaningful differences in the association between TMB and outcome of men and women treated with anti-PD1 or anti-PD-L1 drugs.

The role of TMB as biomarker to select patients who benefit the most from anticancer immunotherapy is still debated, since conflicting results on its predictive value for survival benefit have been reported in trials testing ICIs.^41^

Our analysis suggested that TMB could have a strong and linear association with both PFS and OS in women but not in men, and that considering different TMB cut-off points in men and women may improve its predictive value for both.

All this can potentially help to understand the reason of conflicting results of TMB predictive value observed across trials, that could be due, at least in part, to different ratios of men and women included in the different trials.

This result further corroborates and in turn is potentially explained by the other findings reported in this work. Indeed, the non-linearity of the association between TMB and prognosis of male patients treated with anti-PD-1/PD-L1 drugs, and in particular the observation that treatment benefit started only above a high TMB-threshold, can be due to the less efficient neoantigens presentation to the immune-system observed in tumors of men.

Such our results are consistent with those of a previous work, demonstrating that the TMB’s predictive value for overall response rate (ORR), was significantly higher for females as compared with male patients with advanced NSCLC treated with ICIs.^42^

Notably, we only analyzed data from patients treated with anti-PD1/PD-L1 drugs given as monotherapy and therefore our results could not be valid for other immunotherapy strategies, including anti-CTLA4 drugs given alone or combined with anti-PD1/PD-L1 antibodies, or the combination of chemotherapy with ICIs.

A limitation of this analysis was represented by the fact that we did not have data on previous or subsequent lines of treatment, possibly received by patients analyzed.

Yet, this paper has limitations and further studies are needed to better explore such complex issue.

The main limitation is that only 2.4% of cases assessed (62) were in stage IV. Since almost all patients analyzed had early-stage disease, our conclusions should not be applied beyond this context. However, we expect that sex-based dimorphism of anticancer immune response and immune evasion mechanisms could be even deeper in advanced tumors, as a consequence of continuous immune-editing process and tumor evolution during tumor progression.

Another limitation is the fact that EGFR and ALK gene mutational status was known only for a subgroup of samples analyzed. However, since the expected frequency of such alterations is quite low in non-squamous tumors and almost zero in squamous NSCLC, it is unlikely that the small number of samples that were either EGFR or ALK mutated and with gene mutational status unknown, substantially affected the results, considering the large number of samples and datasets analyzed.

We also did not study other tumor histotypes, while our previous data showed large sex-based differences of ICIs effectiveness also in solid tumors other than NSCLC, including melanoma.

Analyses are ongoing, and our preliminary data suggest sex-based differences in molecular mechanisms of anticancer immune response also in advanced-stage tumors as well as in histotypes other than NSCLC.

In conclusion, data reported here and in our previous works, provided a proof of concept of the importance of the features of the immune system of the host, in shaping the immune response against cancer.

This could have several straightforward implications in the context of both translational and clinical research.

These include the need to explore differential therapeutic approaches and predictive biomarkers in men and women with cancers to improve results for both.

## Supplementary Material

Methods

supplementary fig. 1

supplementary fig. 2

supplementary table 1

supplementary table 2

supplementary table 3

## Figures and Tables

**Table 1:** Main features of each dataset included in the analyses

Variable	Level	LCE-project datasets							ALL LCE-project datasets N=2575	TRACERx dataset
		TCGA LUAD N=515	TCGA LUSC N=501	ROUSSEAUX N=293	SATO N=275	SCHABATH N=442	SHEDDEN N=442	NORO N=107		
**Type of molecular data**		Single-region bulk RNA-sequencing								Multiregion bulk RNA-seq Multiregion WES
**Analyses performed**		Cell-types deconvolution, T-cells phenotype characterization, TIDE-score assessment,GSEA of MSigDB and hypoxia signatures								Cell types deconvolution, TCR repertoire, HLA-LOH, clonal and subclonal TMB, antigen presentation machinery alterations
**Gender, N (%)**	Male	238 (46.2)	371 (74.1)	250 (85.3)	148 (53.8)	202 (45.7)	223 (50.5)	96 (89.7)	1528 (59.3)	62 (62)
	female	277 (53.8)	130 (25.9)	43 (14.7)	127 (46.2)	240 (54.3)	219 (49.5)	11 (10.3)	1047 (40.7)	38 (38)
**Age(years), N (%)**	≤60	157 (30.5)	108 (21.6)	130 (44.4)	91 (33.1)	70 (15.8)	147 (33.3)	29 (27.1)	732 (28.4)	Median: 68
	61-70	174 (33.8)	195 (38.9)	96 (32.8)	91 (33.1)	152 (34.4)	164 (37.1)	62 (57.9)	934 (36.3)	Range: 34-85
	>70	165 (32.0)	189 (37.7)	66 (22.5)	87 (31.6)	199 (45.0)	131 (29.6)	16 (15.0)	853 (33.1)	
	Unknown	19 (3.7)	9 (1.8)	1 (0.3)	6 (2.2)	21 (4.8)	0	0	56 (2.2)	
**Smoking status,**	Smoker	0	0	0	244 (88.7)	335 (75.8)	0	0	579 (22.5)	
**N(%)**	Current Smoker	119 (23.1)	133 (26.5)	0	0	0	32 (7.2)	54 (50.5)	338 (13.1)	40 (40)
	Former Smoker	307 (59.6)	338 (67.5)	0	0	0	268 (60.6)	50 (46.7)	963 (37.4)	48 (48)
	Non-Smoker	75 (14.6)	18 (3.6)	0	28 (10.2)	33 (7.5)	49 (11.1)	3 (2.8)	206 (8.0)	12 (12)
	Unknown	14 (2.7)	12 (2.4)	293 (100.0)	3 (1.1)	74 (16.7)	93 (21.0)	0	489 (19.0)	0
**Race, N (%)**	White	388 (75.3)	349 (69.7)	0	244 (88.7)	399 (90.3)	294 (66.5)	0	1674 (65.0)	97 (97)
	Black	52 (10.1)	30 (6.0)	0	16 (5.8)	13 (2.9)	12 (2.7)	0	123 (4.8)	0
	Asian	8 (1.6)	9 (1.8)	0	7 (2.5)	3 (0.7)	6 (1.4)	0	33 (1.3)	0
	Other	1 (0.2)	0	0	8 (2.9)	2 (0.5)	1 (0.2)	0	12 (0.5)	3 (3)
	Unknown	66 (12.8)	113 (22.6)	293 (100.0)	0	25 (5.7)	129 (29.2)	107 (100.0)	733 (28.5)	0
**Stage, N (%)**	I/II	402 (78.1)	409 (81.6)	226 (77.1)	182 (66.2)	334 (75.6)	371 (83.9)	107 (100.0)	2031 (78.9)	86 (86)
	III	84 (16.3)	84 (16.8)	53 (18.1)	86 (31.3)	63 (14.3)	68 (15.4)	0	438 (17.0)	14 (14)
	IV	27 (5.2)	7 (1.4)	5 (1.7)	6 (2.2)	17 (3.8)	0	0	62 (2.4)	0
	Unknown	2 (0.4)	1 (0.2)	9 (3.1)	1 (0.4)	28 (6.3)	3 (0.7)	0	44 (1.7)	0
**Subtype, N (%)**	Non-Squamous	515 (100.0)	0	293 (100.0)	275 (100.0)	442 (100.0)	442 (100.0)	0	1967 (76.4)	68 (68)
	Squamous	0	501 (100.0)	0	0	0	0	107 (100.0)	608 (23.6)	32 (32)

## Statement of Translational Relevance

It is well known that sex (i.e., the biological differences between men and women) and gender (i.e., behavioral differences associated with being male or female) are variables that affect immune responses to both foreign and selfantigens.

Such sex- and gender- based dimorphism of immune system function, in turn reflects complex interactions between genes, hormones, the environment, and commensal microbiome composition.

In our previous works, we showed that patients’ sex is significantly associated with effectiveness of immune-checkpoints inhibitors (ICIs) in patients with several solid tumors, including NSCLC.

Here we identified meaningful differences in molecular mechanisms that drive anticancer immune response as well as in immune evasion mechanisms exploited by NSCLCs arising in men and women.

Importantly, we showed that all the findings reported, were not related to other variables potentially associated with sex such as patients’ age, stage of disease, tumor histotype and smoking status.

The findings reported in this our work explain our previous clinical observations and can open this area to different immunotherapy strategies in males and females with NSCLC to further improve prognosis of both.

## Abbreviations

aDC: Activated dendritic cells
CD4+ Tcm: CD4+ central memory T- cells
CD4+ Tem: CD4+ effector memory T-cells
CD8+ Tcm: CD8+ central memory T-cells
CD8+ Tem: CD8+ effector memory T-cells
cDC: Conventional dendritic cells
CLP: Common lymphoid progenitors
CMP: Common myeloid progenitors
DC: Dendritic cells
GMP: Granulocyte-macrophage progenitors
HSC: Hematopoietic stem cells
iDC: Immature dendritic cells
ly Endothelial cells: Lymphatic endothelial cells
MEP: Megakaryocyte-erythroid progenitors
MPP: Multipotent progenitors
MSC: Mesenchymal stem cells
mv Endothelial cells: Microvascular endothelial cells
NKT: Natural killer T-cells
pDC: Plasmacytoid dendritic cells
Tgd cells: Gamma delta T- cells
Th1 cells: Type 1 T-helper cells
Th2 cells: Type 2 T-helper cells
Tregs: Regulatory T-cells
