# Supplementary material for: Sex-based dimorphism of anticancer immune response and molecular mechanisms of immune evasion
Source: Clin Cancer Res. Author manuscript; Available in PMC 2021 Aug 4. (PMC7611463; doi:10.1158/1078-0432.CCR-21-0136)
Supplement: Methods [file EMS127617-supplement-Methods.docx]

**Materials and methods**

Source data.

We analyzed public datasets containing data on genome-wide transcriptome analysis of NSCLC samples, from the Lung Cancer Explorer (LCE) project ^6^.

LCE-project, provides the largest collection of NSCLC gene expression datasets, reprocessed to be suitable for computational meta-analysis.^6^

Description of the procedures adopted for reprocessing and normalizing expression data, quality control assessment and standardization of the datasets, has been previously reported.^6^

For our analyses, we focused on the largest datasets on adenocarcinoma and squamous cell carcinoma available in the LCE-project: we included

in the analysis all the datasets with data on more than 250 tumor samples and at least 25 samples from female patients for adenocarcinoma and/or

more than 100 tumor samples and at least 10 samples from female patients for squamous-NSCLC.

For each dataset, patients’ epidemiological and clinical data were available including tumor histology, patient demographics, stage at diagnosis and smoking status.

We also analyzed Whole Exome Sequencing (WES) data of 327 tumor regions from 100 patients with NSCLC and RNAseq data of a subset of 164 tumor regions from 64 tumors, included in the TRACERx lung study (https://clinicaltrials.gov/ct2/show/NCT01888601).

More details on the patient cohort enrolled in the TRACERx lung study have been previously reported.^7-9^

All the analyses conducted on the TRACERx dataset, explored elements of immune response that are known to be of paramount importance for anticancer immune response and in mechanisms of immune evasion used by tumors, but were not evaluable on LCE-project datasets, because they

required multiregion WES and/or RNA-seq data.

Finally, we analyzed data of two independent cohorts of patients with advanced NSCLC treated with anti-PD1 and/or anti-PD-L1 monotherapy

and for which individual patient data (IPD) were available on tumor mutational burden (TMB) and patients’ outcome. ^10-11^

More details on these two cohorts have been previously reported, and were represented respectively by patients included in the MSKCC database as well as by patients enrolled in the POPLAR and OAK randomized clinical trials (RCTs).^10-11^

Computational and statistical analyses.

*1. Assessment of sex-based differences in cell type composition of the immune infiltrate and expression levels of immune checkpoint molecules in the tumor microenvironment (TME)*.

Gene-expression data were analyzed through the previously validated xCell algorithm, to estimate the abundance of different cell types in the microenvironment of each tumor sample included in the datasets of the LCE-project as well as of the TRACERx lung study.

The entire pipeline of xCell has been previously described. ^12,13^

Briefly, xCell, is a computational algorithm that integrates single sample gene set enrichment analysis (ssGSEA) with deconvolution approach,

and permitting estimation of the cell-type composition of the TME, evaluating the expression levels of a compendium of 489 gene signatures that identify 64 different cell types, including multiple adaptive and innate immunity cells, hematopoietic progenitors, epithelial cells, and extracellular matrix cells.^12^

We selected the xCELL algorithm, because it allows to identify the largest number of cell types in TME as compared with other similar algorithms available. Furthermore, the xCELL algorithm was validated using extensive in-silico simulations and also cytometry immunophenotyping, and was shown to outperform several other similar algorithms. ^12^

Gene signatures associated with each cell type are reported in the supplementary table 1.

For each single dataset, mean values of enrichment score (ES) for the 64 different cell-types were calculated in tumors of men and women and then compared using a multivariable linear regression model adjusted for patient age, stage at diagnosis, tumor-histotype and smoking status.

We then performed a meta-analysis of the adjusted sex-related differences obtained in each single dataset using a random-effects model. The false discovery rate (FDR) was used to correct for multiple comparisons. Pooled estimate higher than 0 indicated a greater ES in females, and lower than 0 a greater ES in males.

Gene-expression data from the LCE-project datasets were analyzed through the GSEA method proposed by Subramanian et al.^13^ , using the

following gene sets collection:

1) C5 collection of the Molecular Signatures Database (MSigDB) v 6.

MsigDB is one of the most comprehensive database of gene sets for performing GSEA curated by the Broad Institute of MIT.

The MsigDB C5 collection v6, includes 5917 gene sets derived from Gene Ontology terms belonging to one of the three root GO ontologies - biological process (BP), cellular component (CC), or molecular function (MF) - allowing to comprehensively assess all biological processes, molecular functions and components of cells

2) 16 specific gene signatures, recently defined through single-cell RNA sequencing characterization of the T-cells landscape of NSCLC.

Each GS is associated with a different T-cell subpopulation, characterized by specific functional state and phenotype, including CD8+ and CD4+ naïve T-cells, effector T-cells, pre-exhausted and terminally exhausted. T-cells, and T-cell subpopulations with intermediate functional states as well as T regulatory Cells. (obtained from Guo et al ;supplementary table 1).^14^

3) Two different, previously validated signatures including 26 and 24 genes upregulated in hypoxic TME, obtained respectively from Buffa et al and Yang et al^15-17^

GSEA was originally proposed to evaluate microarray data at the level of gene set to determine whether the gene set(s) analyzed is(are) correlated with the phenotypic class of interest.^13^ We modified the original methodology calculating first sex-related differences for each individual gene in each dataset, adjusted through a multivariable linear regression model for patient age, stage at diagnosis, tumor-histotype and smoking status.

Then, we applied a random-effects model to estimate the overall strength of the association between each gene and sex. The GSEA method using the three GS collection, was then applied on the meta-analytic T-test statistics. When we encountered a gene present in only one dataset, in the GSEA method we used the simple T-test statistic for that gene. Gene labels were permuted to estimate the FDR-corrected statistical significance of the NES. ES (and NES) higher than 0 indicated a greater enrichment of the gene set in females, and lower than 0 a greater enrichment in males.

Both meta-analysis and GSEA were implemented and performed in SAS software v. 9.4 (SAS Institute, Cary, NC).

Finally, a curated list of 78 genes with a key role in anticancer immune response was derived from Thorson et al^18^, and the expression levels of each gene were compared to assess differences between tumors of male and female patients in each single dataset using a multivariable linear regression model adjusted for patient age, stage at diagnosis, tumor-histotype and smoking status.

We then performed a meta-analysis of the adjusted sex-related differences obtained in each single dataset using a random-effects model

and we corrected for multiple comparisons with FDR

*2. Assessment of sex-based differences in mechanisms of immune evasion*

Gene-expression data from LCE-project datasets were analyzed through the validated Tumor Immune Dysfunction and Exclusion (TIDE) tool, that permits quantification of the activation status of two major mechanisms of immune-evasion exploited by tumors: the induction of T-cell dysfunction (T-cell dysfunction mechanism) and the inhibition of T-cell infiltration into TME (T-cell exclusion mechanism).^19^

All the details on procedures implemented to develop and validate TIDE have been previously reported. ^19^

Briefly, TIDE have been developed integrating and modeling data of both patients’ survival and tumor gene expression profiles, from 189 human cancer studies, comprising a total of 33,197 samples.

The T-cell dysfunctional signature was derived identifying genes that influence the function of cytotoxic T cells (CTL) on patient survival outcome in cancer genomics data cohorts analyzed.

The Cox-PH survival regression was applied to test how the level of CTL interacts with other genes in the tumor to affect patients’ survival outcome. The linear model Hazard = *a*×CTL + *b*×*V* + *d*×CTL×*V* + *c* was solved using the Cox-PH regression. The CTL level is estimated through the bulk-tumor expression average of *CD8A*, *CD8B*, *GZMA*, *GZMB* and *PRF1*. In the Cox-PH model, the death hazard was estimated through the patient survival information. The variable *V* represents the expression level of a candidate gene in the test. Since CTL correlates with favorable survival outcome, the coefficient *a* is always negative. The association slope between CTL and Hazard is *a* + *d*×*V.*  If the coefficient *d* is positive, a higher *V* level will flatten the slope between CTL and Hazard, indicating a reduced association between the cytotoxic T cell level and better survival outcome. If *d* is negative, a higher *V* level will sharpen the slope between CTL and Hazard, indicating an increased association between the cytotoxic T cell level and better survival outcome. The T cell dysfunction score for each gene is defined as the Wald test *z* score, which is the coefficient *d* divided by its standard error. The resulting T cell dysfunctional signature is a genomewide vector, where the *z* score of each gene is the interaction coefficient *d* divided by its standard error. Genes with significant *z* scores are not restricted to genes expressed by T cells but could be expressed in cancer cells or different immune cells associated with T cell dysfunction

The T cell exclusion signature was derived modelling a genome-wide expression signature with a strong negative correlation with the CTL levels across tumors

For each tumor sample we calculated two scores, the “T-cell dysfunction score” and “T-cell exclusion score”: both scores range from -4 to +4, with the higher score levels being associated with greater activation status of the corresponding mechanism of immune-evasion.^19^

For each single dataset, mean values of the “T-cell dysfunction score” and “T-cell exclusion score” were calculated in tumors of men and women and then compared using a multivariable linear regression model adjusted for patient age, stage at diagnosis, tumor-histotype and smoking status.

We then performed a meta-analysis of the adjusted sex-related differences obtained in each single dataset using a random-effects model. The Q test was performed to assess between-study heterogeneity, and the I^2^ statistics, which express the percentage of the total observed variability due to heterogeneity, were also calculated

A pooled-estimate score higher than 0 indicated a greater activation status in females of the corresponding mechanism of immune-evasion, and lower than 0 a greater activation in males.

*3. Assessment of sex-based differences in TCR repertoire diversity, tumor neoantigens load and alterations in neoantigens presentation machinery.*

Multiregion Bulk RNAseq and WES data from TRACERx lung study were employed to assess in each tumor the TCR repertoire diversity, number of predicted neoantigens and their clonal distribution , occurrence

of loss of heterozygosity (LOH) events at the HLA class I locus, as well as genetic disruptive events in other antigen presentation pathway genes.

Assessment of T-cell receptor abundance and entropy score:

A previously developed quantitative experimental and computational T-cell receptor (TCR) sequencing pipeline was used for the high-throughput sequencing of α and β TCR chains. TCR sequencing was performed on whole RNA extracted from multi-region tumor specimens.^17^ A distinct feature of this TCR sequencing protocol is the use of a unique molecular identifier that enables correction for PCR and sequencing errors, thereby providing a quantitative and reproducible method of library preparation. ^20,21^

For each tumor region, the Shannon diversity was estimated using the command ‘entropy.empirical’ from the ‘entropy’ R package. This was calculated on the basis of the number and prevalence of different TCR subclones found in that region, such that a tumor region that contained only one subclone was assigned a value of 0.

The Shannon diversity score, *H*, followed the formula *H* = −Σ*p_i_* × log(*p_i_*), in which *p_i_* is the probability of the *i*th clone appearing in the tumour cell population.

Assessment of ubiquitous expanded TCRs in tumor

We counted the number of TCRs detected with frequencies above a range of frequency thresholds in the tumor samples. To focus on the most expanded TCRs, we examined those present above a threshold frequency of 2/1,000 (corresponding to the top 1% of the empirical TCR frequency distribution) in at least one region of the tumor.^8,9^

We calculated the relative abundance of the TCR in the tumor (averaged over all tumor regions) from the same patient.

Expanded intratumoral TCRs were subsequently classified as ubiquitous or regional, as previously described.^8,9^

In particular, expanded TCRs were classified as regional if they were absent from at least one region of the tumor and as ubiquitous if otherwise. Ubiquitous TCRs can be absent from the data for specific regions, but this should be attributed to sampling rather than true spatial heterogeneity. ^8,9^

For this analysis, we analyzed tumors with TCR data for at least three different regions.

Assessment of predicted neoantigen load:

Novel 9–11mer peptides that could arise from identified non-silent mutations present in the sample were determined.^7^ The predicted half-maximal inhibitory concentration binding affinities and rank percentage scores — representing the rank of the predicted affinity compared to a set of 400,000 random natural peptides — were calculated for all peptides that bound to each of the patient’s HLA alleles using netMHCpan-2.8. and netMHC-4.0.^22,23^ Using established thresholds, predicted binders were considered to be those peptides that had a predicted binding affinity <500nM or a rank percentage score <2% by either tool. ^22,23^

When RNA-seq data were available, a neoantigen was considered to be expressed if at least five RNA-seq reads mapped to the mutation position, and at least three contained the mutated base. ^22,23^

Assessment of HLA LOH and/or alteration in other antigen-presentation-pathway genes:

Tumor samples that contain an HLA LOH event were identified using a previously validated method.^8^

Antigen-presentation-pathway genes were compiled from a previous report, and affected the HLA enhanceosome, peptide generation, chaperones or the MHC complex itself.^24^

For each tumor sample, we assessed disruptive events (i.e. non-silent mutations or copy-number loss defined relative to ploidy) of the following genes: CIITA, IRF1, PSME1, PSME2, PSME3, ERAP1, ERAP2, HSPA (also known as PSMA7), HSPC (also known as HSPBP1), TAP1, TAP2, TAPBP, CALR, CNX (alias CANX), PDIA3 and B2M.

*4. Evaluation of sex-based differences in the association between Tumor Mutational Burden (TMB) and outcome of patients treated with anti-PD1 or anti-PDL1 drugs.*

We analyzed data from MSKCC dataset on patients treated with anti-PD1 or anti-PD-L1, to assess the association between tissue-TMB (tTMB) and OS according with patients’ sex.

Data of patients treated with the anti-PDL1 atezolizumab in the OAK and POPLAR RCTs, were evaluated to assess the association between

blood-TMB (bTMB) and PFS according with patients’ sex.

We did not explore sex-based differences in the association between bTMB and OS, since in the original analysis performed on the whole population a significant predictive value of bTMB was reported for PFS but not for OS.^11^

For all the analyses reported, the tissue and blood TMB were analyzed as a continuous variables. Wilcoxon rank-sum test was used to compare the distribution of tissue and blood TMB between female and male patients. Cox proportional hazard regression model was used to evaluate the association between tTMB and bTMB and patient OS and PFS respectivelly. Male and female subgroups were analyzed separately.

Departure from linearity in the relationship between tissue or blood TMB and the hazard of death was investigated with restricted cubic spline (RCS) models with four knots located at the 20^th^, 40^th^, 60^th^ and 80^th^ percentiles of the TMB distribution of female and male patients, respectively.^25^ The likelihood ratio test was used to determine whether the RCS model significantly increased the likelihood function compared with a simpler model that assumed a linear relationship.

Multivariable analyses were performed excluding patients with tumors harboring EGFR gene mutation or ALK gene translocation and considering only those patients with available data on the following adjustment factors: age, smoking history (i.e., never versus previous or current smoker), tumor histotype (i.e., adenocarcinoma, squamous carcinoma or other) and type of specimen analyzed (i.e., primary tumor versus metastasis), number of metastatic sites at enrollment, sum of longest diameter of target lesions at baseline, and PD-L1 expression levels (TC3IC3 vs TC0/1/2IC0/1/2).

Statistical analysis were performed with SAS software v. 9.4 (SAS Institute, Cary, NC) and R software (version 3.4.1).
