# Supplementary figures and images for: Sex-based dimorphism of anticancer immune response and molecular mechanisms of immune evasion
Source: Clin Cancer Res. Author manuscript; Available in PMC 2021 Aug 4. (PMC7611463; doi:10.1158/1078-0432.CCR-21-0136)

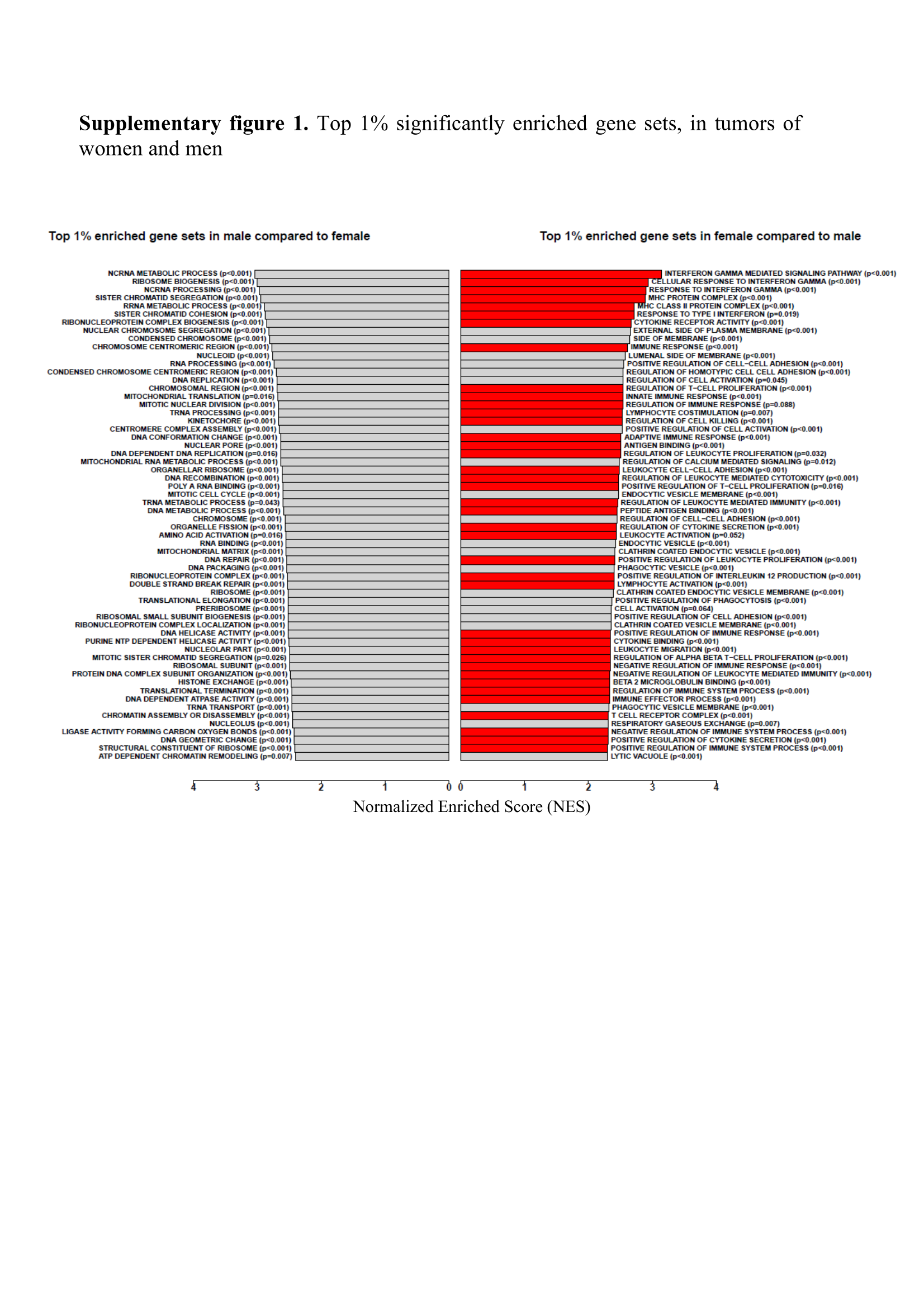

Supplement: supplementary fig. 1 [file EMS127617-supplement-supplementary_fig__1.tiff]

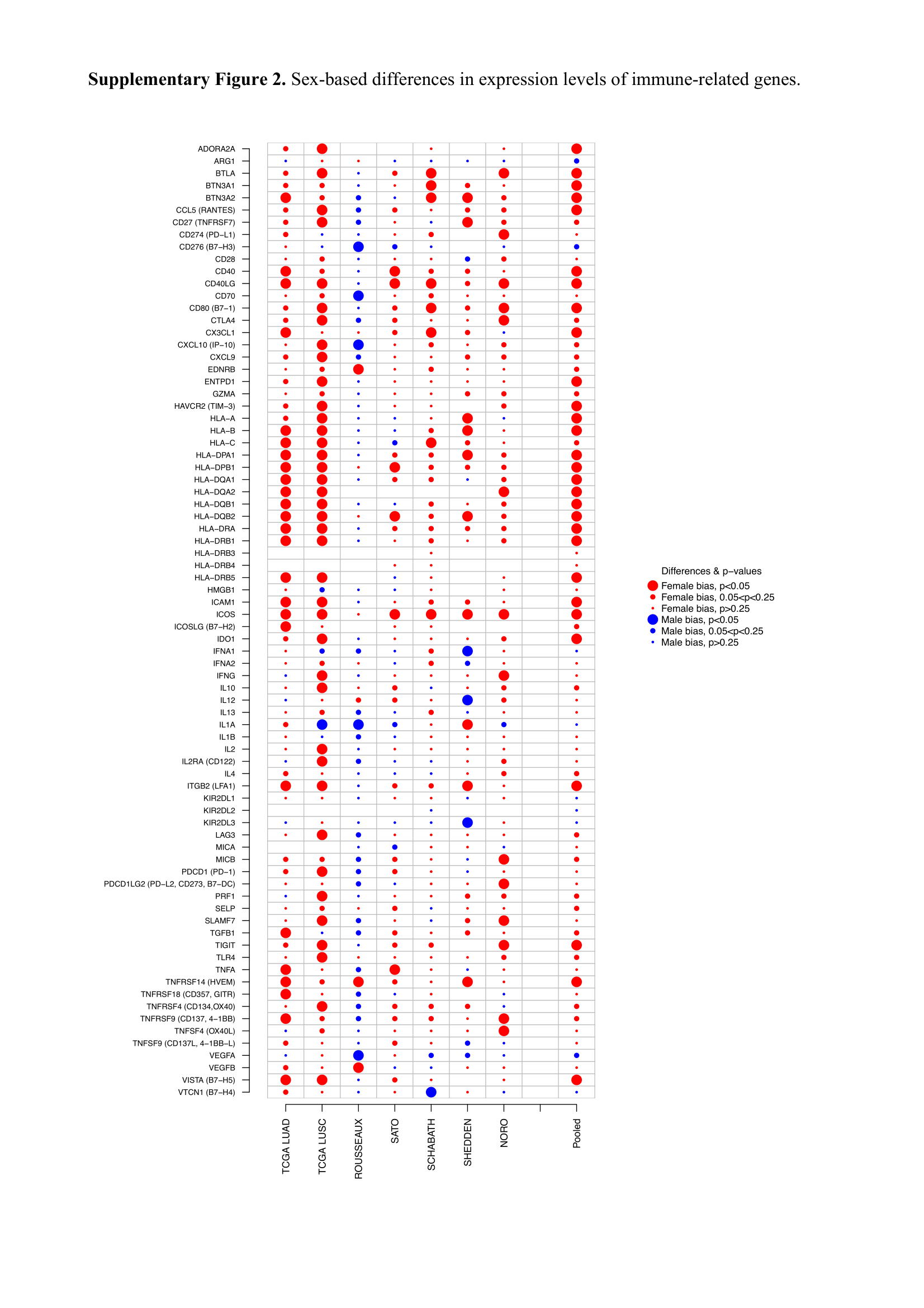

Supplement: supplementary fig. 2 [file EMS127617-supplement-supplementary_fig__2.tiff]
